# Medical Books

**Published:** 1867-10

**Authors:** 


### *Medical Books.*

Messrs. S. C. Griggs & Co. (39 and 41 Lake Street) have favored us with a catalogue of medical books which they have for sale, with accompanying price list. Students or practitioners can obtain the same free, on application. They have a large and varied assortment, and offer special inducements to purchasers. The editorial pen itches to say one thing more about this firm, but temporarily forbears.

